# Supplementary figures and images for: Identification of hub genes and small-molecule compounds related to intracerebral hemorrhage with bioinformatics analysis
Source: PeerJ. 2019 Oct 25;7:e7782. doi: 10.7717/peerj.7782 (PMC6816389; doi:10.7717/peerj.7782)

A

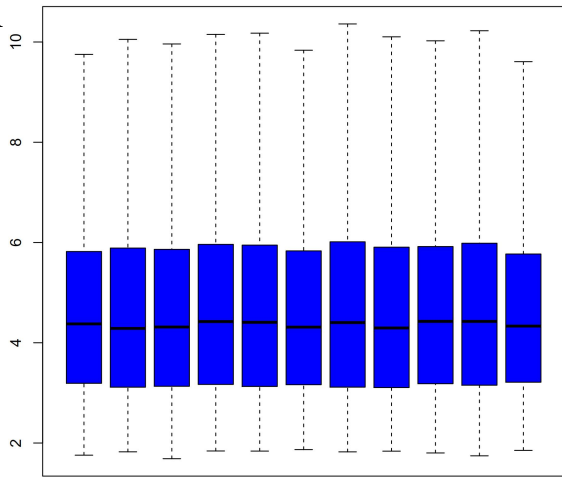

B

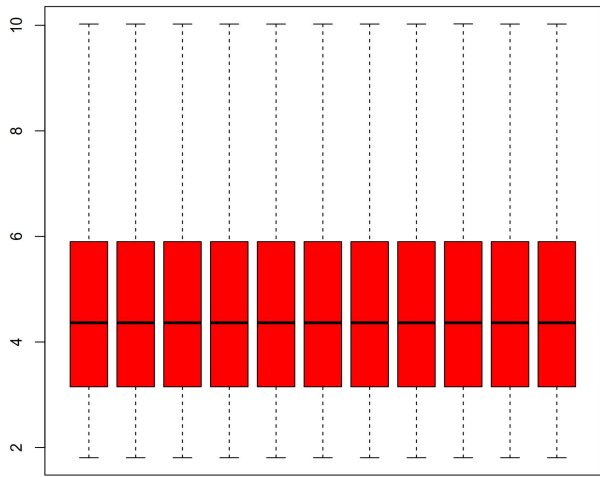

Supplement: Figure S1 — (A) Blue indicates that the raw data has not been standardized. (B) Red indicates that the raw data has been normalized. [file peerj-07-7782-s002.pdf]
