# Supplementary material for: Identification of hub genes and small-molecule compounds related to intracerebral hemorrhage with bioinformatics analysis
Source: PeerJ. 2019 Oct 25;7:e7782. doi: 10.7717/peerj.7782 (PMC6816389; doi:10.7717/peerj.7782)
Supplement: Table S1 [file peerj-07-7782-s003.doc]

**Supplemental table 1**

| Term | Primer Sequence (5´-3´) |
| --- | --- |
| IL6 | F:GGTCTTCTGGAGTTCCGTTTC  R:AGCATTGGAAGTTGGGGTAG |
| TLR2 | F:GGGACTCACAGCAAACACAA  R:CTGAACCAGGAGGAAGATAAACTA |
| CXCL1 | F:AAGGGCGGAGAGATGAGAGT  R:CRAGAAGGAGCATTGGTTAAAG |
| TIMP1 | F:TGGCATCCTCTTGTTGCTATC  R:ACAGCGTCGAATCCTTTGAG |
| PLAUR | F:AGGCTTAGATGTGCTGGGAAAC  R:CAGGGAGGCAATGAGGATAA |
| SERPINE1 | F:GCAACAAGAGCCAATCACAA  R:CGTAGGGAGAGAAGACCACATT |
| SELE | F:ACATCTGGTGGCGATTCAG  R:GTCCTCGTTTCTTTGTTTGTTG |
| CCL4 | F:CCCAGCCAGCTGTGGTATTT  R:ACATACTCATTGACCCAGGGC |
| CCL20 | F:CATCACTGCAGACCTGATTT  R:AGCGCCCTTCATATTGTG |
| CD163 | F:GGCCCATGTGGACAGATAGT  R:CATGCAACCTCAGCATCATT |
| GAPDH | F:ATGCCGCCTGGAGAAACC  R:GCATCAAAGGTGGAAGAATGG |
